# Frontiers in pharmaceutical nanotechnology

**DOI:** 10.3762/bjnano.10.244

**Published:** 2019-12-17

**Authors:** Matthias G Wacker

**Affiliations:** 1 National University of Singapore, Faculty of Science, Department of Pharmacy, 6 Science Drive 2, 117546 Singapore

**Keywords:** drug delivery, nanocarriers, nanomedicines, nanotheranostics, pharmaceutical nanotechnology

Today, pharmaceutical nanotechnology is a very dynamic and evolving research area that integrates a wide variety of disciplines such as chemical, biological and biomedical science. At the frontier of knowledge, nanoparticles, exosomes and even more advanced drug delivery systems [1] blur the line between drug discovery and formulation science. They have fundamentally changed our understanding of the way dosage forms can facilitate drug therapy.

Prof. Jörg Kreuter has been a pioneer in this research area and dedicated his life’s work to nanoparticle research and the blood–brain barrier [2]. One of his most outstanding discoveries, the active transport of nanoparticles into the central nervous system using the low-density lipoprotein receptor family [3–6], provided an entry route for the cytostatic drug doxorubicin into the brain. The drug delivery system has been tested in a phase II clinical trial and hopefully will make its way into the market.

Although nanotechnology has gained significant attention in the scientific community, this is one of very few examples where targeted delivery was successfully developed to the stage of clinical translation. Today, a fierce competition between different technologies decides on commercial success, and consequently, the number of new therapies [7].

Nanotechnology comes in a thousand varieties and there is a rising number of “engineered nanomaterials” under development [1,8]. In this dedicated issue, we present some of the latest trends ranging from the synthesis of new materials [9] to the application of nanoparticles in our fight against drug resistance [10]. But what are the frontiers of tomorrow?

Recently, the Center for Drug Evaluation and Research within the United States Food and Drug Administration presented the progress in new drug applications and concluded that, although the use of nanomaterials has generally increased, the number of approved nanomedicines is still very limited when compared to the tremendous research activity in this area [1].

Because nothing is older than yesterday’s newspaper, this editorial will take a look into the crystal ball.

Nanomedicine was proclaimed to revolutionize medicine, but what we are seeing at present is a slow transformation rather than a revolutionary overthrow. Now more than ever, the potential for clinical translation will be in the spotlight. In many areas, nanotechnology is already accepted, for example, in the production of nanocrystals. To date, the nanomilling platform of Elan Drug Technologies is widely used for the formulation of poorly soluble compounds [11]. Also, with regards to the topical administration route, nanometer-scale excipients have been rarely associated with safety issues and have been widely applied in the development of semisolids. But we still have much to learn.

Pharmaceutical science has indisputably become more complex with the discovery of nanocarrier-based delivery systems. Fueled by first successes in the 1990s, liposomes were at the forefront of cancer therapy [12–13]. The challenges associated with their characterization earned them the name “non-biological complex drugs” (NBCDs) [8].

Nowadays, there is broad acceptance for liposomal drugs as a niche product, but we still do not know much about the attributes that enable targeted delivery in humans [14]. In 2018, Alnylam announced the approval of a first-of-its-kind RNA interference (RNAi)-based drug, Onpattro™, which uses solid lipid nanoparticles to protect the sensitive compound from early degradation. Again, lipid materials rather than synthetic polymers have been used for drug delivery applications.

In pharmaceutical research, the economic success of drug products is closely related to product efficacy and safety. While lipids have a solid track record of clinical safety [7,12], there are few polymers generally used for the parenteral route of administration. Further, the procedure of drug approval usually does not provide market exclusivity or a general recommendation for the excipient but for the drug product only [7]. This together makes it more difficult to justify the time-consuming and expensive toxicological studies required to establish clinical safety of the excipient. Consequently, there is a trend towards materials with a long history of medical use [12].

Pharmaceutical nanotechnology is not limited to translational research but, after many years of trial and error, we have to accept that reliable pharmacological and toxicological effects are a key element of industrial formulation development. Understanding the intertwined processes involved in biodistribution of deposition of nanocarrier delivery will finally lead to a broader acceptance, and consequently, to successful translation from bench to bedside.

Matthias G. Wacker

Singapore, November 2019
